# Adrenal Crisis Secondary to Small Bowel Obstruction Caused by a Bezoar

**DOI:** 10.7759/cureus.21498

**Published:** 2022-01-22

**Authors:** Patrícia Campos, Joana Almeida, Maria João Ferreira da Silva

**Affiliations:** 1 Department of Anesthesiology, Intensive Care and Emergency, Centro Hospitalar e Universitário do Porto, Porto, PRT

**Keywords:** small bowel obstruction, shock, acute adrenal insufficiency, bezoar, abdominal pain

## Abstract

Bezoars are aggregates of undigested materials that accumulate in the gastrointestinal lumen. They are a rare cause of small bowel obstruction and are mostly diagnosed in patients with small bowel disease. Patients with panhypopituitarism are more susceptible to developing metabolic and haemodynamic instability, particularly during perioperative period.

We present the case of a male patient with small bowel obstruction secondary to a bezoar. The patient was admitted to the hospital due to upper abdominal pain and emesis, presenting with clinical signs of shock. He had a history of iatrogenic panhypopituitarism and had been submitted to a bilateral inguinal hernioplasty 15 days before. Abdominopelvic computed tomography with angiography revealed small bowel obstruction secondary to a bezoar. Stress-dose hydrocortisone was administered to treat the underlying haemodynamic instability, followed by exploratory laparotomy. The bezoar was removed and eventually the patient recovered with a tapering regimen of hydrocortisone.

The diagnosis of small bowel obstruction secondary to bezoar can be challenging. The shock could be related to an adrenal crisis precipitated by the bezoar in the setting of increased susceptibility due to the recent surgery.

## Introduction

Bezoars are composed of undigested materials that accumulate in any part of the gastrointestinal tract, the stomach being the most common location [1,2]. It is a rare cause of small bowel obstruction, accounting for 0.4%-4% of cases. They are mainly diagnosed in patients with conditions that cause stagnation within a dilated bowel segment such as small bowel diseases, including diverticuli, strictures, tumours, previous gastrointestinal surgery, and gastric motility disorders, such as diabetes, hypothyroidism, pernicious anaemia, and kidney failure [2].

Hypopituitarism is a partial or complete insufficiency of the anterior and posterior lobe of the pituitary gland, leading to decreased or absent secretion of corticosteroids, growth, sexual, and thyroid hormones [3,4]. These patients are prone to develop adrenal crisis with metabolic and haemodynamic instability in conditions associated with acute physiological disturbances, such as the perioperative period [4,5].

Adrenal crisis is a life-threatening emergency defined as an acute deterioration in health status associated with hypotension, that resolves within one to two hours after parenteral glucocorticoid administration [5]. Supplementary glucocorticoid should be administrated for physiological stress, such as major surgery [5].

We present the case of a male patient with a history of panhypopituitarism who presented to the emergency department with haemodynamic shock secondary to the adrenal crisis in the context of small bowel obstruction caused by a bezoar a few weeks after abdominal surgery.

## Case presentation

A 65-year-old Caucasian male was admitted to the emergency department resuscitation room with upper abdominal pain and emesis of one-day duration without guarding. He had a history of panhypopituitarism, dyslipidaemia, and was overweight. The panhypopituitarism was iatrogenic, secondary to a pituitary surgery for non-secretory macroadenoma 17 years ago. He had since been under daily supplementation with glucocorticoid and thyroid hormone replacement along with monthly testosterone supplementation. He had a duodenal surgery for a duodenal ulcer 30 years ago and had been subjected to a bilateral inguinal hernioplasty 15 days before this admission. No perioperative supplementary glucocorticoid had been given.

Physical examination at admission revealed non-measurable arterial pressure with a filiform pulse, sinus tachycardia, and prolonged capillary refill time. He was polypneic with a peripheral O_2_ saturation of 98% on a high concentration mask. Pulmonary and cardiac auscultation were normal. He was noted to have diffuse abdominal tenderness on abdominal palpation, but no signs of peritoneal irritation were evident. The surgical wound had no inflammatory signs. The patient was drowsy but easily aroused, with no focal neurological signs and afebrile. Volume resuscitation with 20 mL/kg of intravenous crystalloid fluid and noradrenaline infusion were initiated to maintain mean arterial pressure > 65 mmHg. Microbiological samples were collected and empirical antibiotic therapy with piperacillin/tazobactam was started.

**Figure 1:**
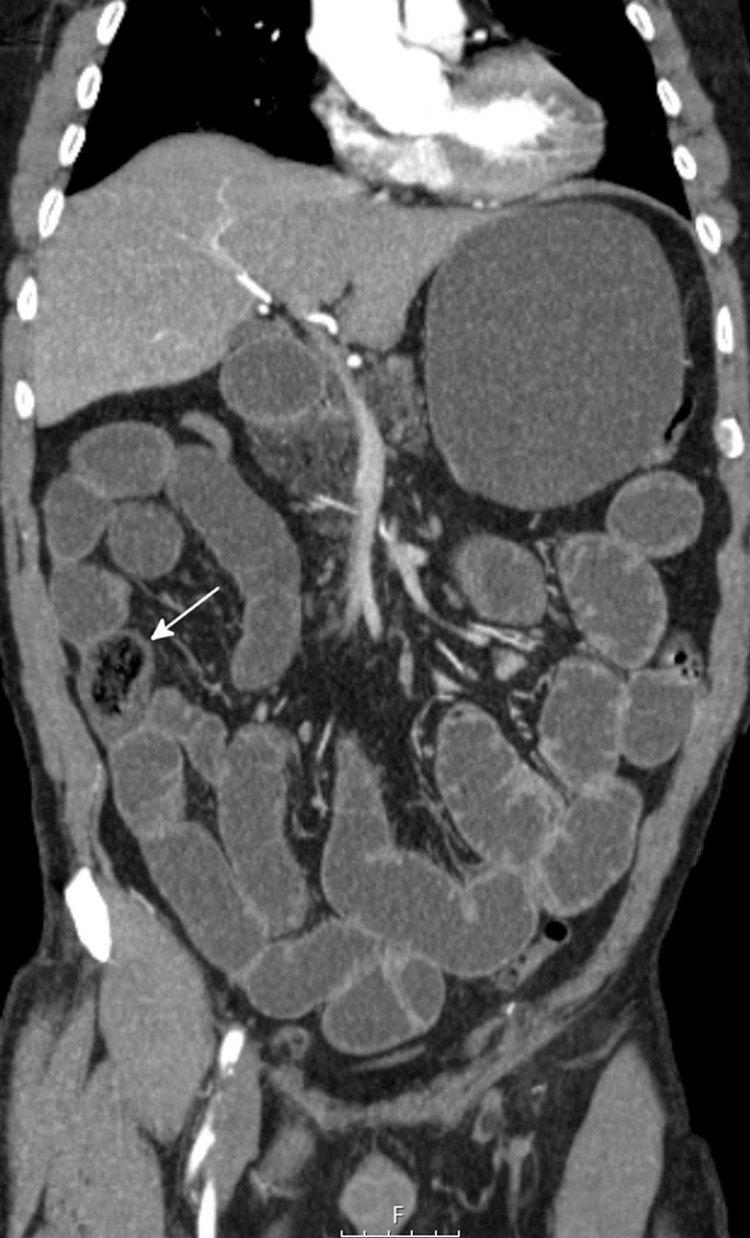
Abdominopelvic computed tomography with angiography scan of the abdomen and pelvis showing an endoluminal nodular image in the distal ileum compatible with a bezoar (arrow).

ST-segment depressions in V2-V5 were visible in the electrocardiogram. Blood analysis revealed metabolic acidosis with hyperlacticaemia (7.8 mmol/L), hypoxemia (PaO_2_/FiO_2_ ratio of 186), acute kidney injury with KDIGO stage 2, lactic dehydrogenase 330 U/L, total creatine kinase 286 U/L, and negative myocardial necrosis markers. No hepatic cytocholestasis or elevation of inflammatory parameters was evident. Abdominopelvic computed tomography with angiography revealed oesophageal, gastric, and small intestinal distension due to water content; a 40-mm endoluminal nodular image with gaseous areas in the distal ileum with the distal collapse of the remaining ileum, and an image of identical characteristics of 20 mm in the proximal ileum (Figure 1).

These radiological findings were compatible with the diagnosis of intestinal bezoar.

Given the patient's history of panhypopituitarism and recent surgical events, the diagnostic hypothesis of adrenal crisis secondary to the bezoar was considered, and an intravenous bolus of 200mg of hydrocortisone was administered. While awaiting exploratory laparotomy in the operating room, the patient improved clinically with complete cessation of vasopressor support and decreasing lactatemia (2.6 mmol/L). During exploratory laparotomy, several intestinal postoperative adhesions were evident. A foreign body with morphological characteristics of a phytobezoar was noted in the distal ileus, 15 cm proximal to the ileocecal valve, with proximal small bowel dilatation. The foreign body was fragmented through extrinsic compression and distal milking was conducted with passage through the ileocecal valve, with no need for partial enterectomy.

During perioperative convalescence, cardio-renal and respiratory dysfunction resolved and the patient had an uneventful recovery in the surgical ward. The stress dose steroids were gradually tapered off and reduced to basal supplementation.

## Discussion

Small bowel obstruction caused by bezoar usually occurs at the narrowest part of the small intestine, most commonly at the distal ileum followed by the jejunum [2].

The clinical manifestations of small bowel obstruction caused by bezoar are difficult to distinguish from intestinal obstruction caused by other factors [2]. The most common presenting symptoms of small bowel obstruction are nonspecific, so the diagnosis is rarely made clinically, which may delay surgical treatment and increase morbidity and mortality [2]. The symptoms may vary according to bezoar sizes or obstruction sites, ranging from asymptomatic to the most common symptoms such as abdominal pain, bloating, nausea, vomiting, and signs of gastrointestinal bleeding [1,2]. Imaging studies are the mainstay for the diagnosis in the early stages [2]. A computerised tomography scan is useful to detect gastric and small intestinal bezoars, especially in the latter when surgical removal is planned. Complications include gastric outlet obstruction, ileus, ulcerations, and subsequent gastrointestinal bleeding, obstruction, and perforation of the gastrointestinal tract, particularly in small intestinal bezoars [1]. Intestinal bezoars are generally surgically removed; it is inevitable in patients presenting with intestinal obstruction, ileus, or refractory bezoars [1].

Our patient presented to the resuscitation room with symptoms suggestive of intestinal obstruction and haemodynamic shock. He had an increased risk of small bowel intestinal bezoar formation due to previous duodenal surgery and postoperative bowel adhesions, which indirectly affects gastrointestinal motility and emptying [2].

We have found no cases in the literature describing shock secondary to a bezoar. We theorize that the shock was secondary to the adrenal crisis precipitated by the bezoar, in a patient with increased susceptibility due to recent surgical stress and iatrogenic panhypopituitarism.

Adrenal crisis is characterized as an acute deterioration in health status associated with hypotension, and concomitant features include acute abdominal pain, delirium, obtundation, hyponatremia, hyperkalemia, hypoglycemia, and pyrexia [5]. It may occur due to inflammation secondary to an infection, nonadherence to glucocorticoid replacement therapy, or secondary to serious acute conditions in which endogenous cortisol production is insufficient, including a surgical procedure or trauma [5,6].

The treatment consists of prompt administration of intravenous hydrocortisone, given as a 100 mg bolus, followed by 200 mg every 24 hours, before prolonged hypotension leads to irremediable effects. Intravenous normal saline should be administered; 5% dextrose in normal saline is reserved for patients with hypoglycemia [7].

The cause of adrenal crisis must be investigated and treated. Persistent shock despite the treatment of adrenal crisis suggests another cause for hypotension [5,7]. Our patient promptly responded to glucocorticoid therapy, supporting the diagnosis of adrenal crisis. The presentation of secondary adrenal crises is usually mild, with no hypoglycemia and electrolyte abnormalities compared with the primary form. Our patient presented without hypoglycemia or typical electrolytical alterations, which is coherent with the diagnosis of adrenal crisis secondary to a bezoar [8].

Since patients with panhypopituitarism are more prone to develop adrenal crisis in the perioperative period, supplementary glucocorticoid should be administrated [4,5]. On the day of surgery, in cases of minor to moderate surgical stress, 25-75 mg of intravenous hydrocortisone per 24 hours for one to two days is recommended; in cases of major surgical stress, 100 mg of intravenous hydrocortisone followed by 200 mg of hydrocortisone per 24 hours should be administered [7].

## Conclusions

The diagnosis of small bowel obstruction secondary to bezoar can be challenging and often delayed due to its rarity. Adrenal crises are sometimes difficult to identify in the emergency department. Various factors can obscure the presentation, delaying or preventing the correct diagnosis, with subsequent poor patient outcomes. It is essential to have high suspicion in patients that receive corticoid supplementation.

This case reports an unusual presentation of a rare clinical practice diagnosis, a bezoar, highlighting the importance of a prompt diagnosis to avoid severe complications of these two potentially life-threatening conditions.
